# Preservation of fatty acid signatures in three vertebrate species after six months of storage at various temperatures

**DOI:** 10.1371/journal.pone.0204207

**Published:** 2018-09-17

**Authors:** Petteri Nieminen, Reijo Käkelä, Tero Mäkinen, Olli Laine, Teemu Takalo, Anne-Mari Mustonen

**Affiliations:** 1 Institute of Biomedicine/Anatomy, School of Medicine, Faculty of Health Sciences, University of Eastern Finland, Kuopio, Finland; 2 Department of Environmental and Biological Sciences, Faculty of Science and Forestry, University of Eastern Finland, Joensuu, Finland; 3 Molecular and Integrative Biosciences Research Programme, Faculty of Biological and Environmental Sciences, University of Helsinki, Helsinki, Finland; 4 Helsinki University Lipidomics Unit, Helsinki Institute for Life Science (HiLIFE), Helsinki, Finland; University of Illinois, UNITED STATES

## Abstract

Fatty acid (FA) signatures (FAS) are important tools to assess the foraging ecology of wild animals. The present study was conducted to assess how well the general FAS and the proportions of individual FA are preserved in fat samples stored at different temperatures (–196, –80, –20, +4 and +20°C). Using three species (laboratory rat, American mink and rainbow trout), FAS were determined immediately upon sampling. Thereafter, eight subsamples per storage temperature from the inner part of the sample unaffected by oxygen and light were re-analyzed after 1, 2, 3, 7, 28, 84 and 168 days. Each time the remaining sample was sealed in its vial after replacing air with nitrogen gas. The results were tested with the mixed model and discriminant analyses. Generally, the FAS were well preserved regardless of storage temperature, and only a few major FA showed significant changes even after the 6-month period at room temperature. After an initial first-day change in proportions, presumably due to post-mortem enzymatic activities, the remaining minor changes could not be clearly attributed to either further autolysis, decomposition or autoxidation. In the discriminant analysis, the species-specific differences dominated and remained distinct even after 6 months. Furthermore, the analysis mostly classified the samples preserved at sub- and above-freezing temperatures separate from each other, and the general deviation from the initial analysis results was present as early as after 1 day. If FAS are to be analyzed in a very precise manner, the analysis should be performed immediately upon sampling. However, FAS remain adequately reliable for long periods of time even without preservation in deep freeze, widening the availability of potential samples for studies on foraging ecology and related disciplines.

## Introduction

Fatty acid (FA) signatures (FAS; relative proportions of individual FA) yield information about the long-term nutrition of animals and humans and have practical applications, for instance, in foraging ecology, where they can be used to examine spatial and temporal differences in diets within and between species [1–4]. FAS can remain relatively stable even for long periods of time when biological samples are stored frozen. It has been reported that storage at –80°C affected the FA composition of human serum only in a minor and inconsistent way [5]. Proportions of major FA remained stable while small increases were detected for minor polyunsaturated FA (PUFA), which did not jeopardize the analysis of samples preserved for up to 10 years. In a similar manner, human blood samples showed only a few small changes in the plasma triacylglycerol (TAG) and erythrocyte phosphatidylcholine fractions after 2–2.5 years of storage at –80°C [6]. Both the above studies suffer from the fact that there were no initial FA analyses upon sampling but the samples were analyzed for the first time only after 0.5–1.5 years. The FA composition of biopsies from human subcutaneous fat also remained stable at –80–+4°C for 1.5 years [7].

In addition to human samples, FAS stability was previously examined regarding frozen food items. It was noticed that after 12 months at –18°C the percentages of total PUFA and the n-3/n-6 PUFA ratios decreased in muscle total lipids of stored fish [8]. The proportions of saturated FA (SFA) also decreased, while those of monounsaturated FA (MUFA) increased at 1–12 months. The changes in FAS were smaller with vacuum packaging suggesting a potential role for PUFA oxidation in the process. In another study, n-3/n-6 PUFA ratios decreased in fish muscle after 12 months of storage at –18°C but SFA sums increased in total lipids [9]. In addition, a retrospective study (1973–2009) was able to distinguish FA patterns of the Baltic herring (*Clupea harengus membras*; all soft tissues included) from different locations in the Baltic Sea at all decades, despite time-related loss of particular n-3 PUFA during storage at –25°C [10]. When samples from Baltic grey seal (*Halichoerus grypus*) blubber, almost pure lipid, were stored at +2°C for up to 6 months, there were no major differences in their general FA profiles [11]. These results suggest that FAS could be resistant to changes during storage, providing benefits in research costs regarding storage in deep freeze and enabling the preservation of samples for longer periods before further processing for FA analyses.

FA and consequently FAS change after sampling due to three principal processes [12–14]. i) Autolysis commences immediately upon death or sampling and it is caused by lysosomal enzymes. Autolysis degrades lipids by lipases and the rate of it depends on the ambient temperature (T_a_). This short-term process can be slowed down, but not totally prevented, with refrigeration or freezing. Actual ii) decomposition of tissues follows autolysis, and lipid degradation is caused by microbial lipolytic enzymes either from the organism or the environment. Similar to autolysis, decomposition is faster at higher T_a_. iii) Autoxidation mostly affects unsaturated FA (*i*.*e*., MUFA and PUFA) and would thus be a potential cause of data loss when analyzing FAS. The more the FA molecule has double bonds, the more prone it is to autoxidation. For example, the rate of autoxidation of 18:3 PUFA compared to 18:0 SFA is approximately 2500:1. Generally, autoxidation takes place due to the presence of oxygen. Oxidation of FA can also be caused by thermal oxidation, enzymatic oxidation and photo-oxidation.

The aim of the present study was to examine the time frame of FAS change during storage at various T_a_, as previous experiments have mainly focused on cold storage. Stability of FAS within tissues for reasonable amounts of time at warmer conditions would enable their determination also from wild animal carcasses, thus, widening the availability of potential samples for studies on foraging ecology and on endangered species. In addition, preservation of FAS during storage could enable the simultaneous analysis of samples collected during a longer period of time. Thus, the present study has significant methodological applications. We hypothesized that i) because of various decay processes, reliable determinations of FAS would become impossible after a certain period of time, that ii) the proportions of PUFA would be more susceptible to change (decrease) during storage and that iii) the changes in the relative amounts of FA would be the least conspicuous at lower storage T_a_. The results showed that the FAS were well preserved at all T_a_, and only a few major FA showed statistically significant but percentually small changes even after the 6-month period at room temperature (T_r_).

## Materials and methods

### Animals and sampling

The study protocol was evaluated by the Animal Experiment Board of Finland and, as no actual research procedures were conducted on live animals, the samplings after euthanasia did not require permits based on the Finnish Act on Animal Experimentation (62/2006). We chose vertebrates that have different FA profiles. White adipose tissue samples were obtained from the subcutaneous abdominal fat of the Sprague Dawley rat (*Rattus norvegicus*; n = 8 males) and the farmed white American mink (*Neovison vison*; n = 8 males) and from the intra-abdominal fat of the sea-farmed rainbow trout (*Oncorhynchus mykiss*: n = 8 females) after standard euthanasia (rats: CO_2_ and decapitation; mink: electrocution; fish: stunning and exsanguination). Mink and fish were commercially farmed animals the euthanasia of which was performed during the regular pelting or harvest season and the sampling did not affect the procedures. The samples were taken into Eppendorf vials that were packed as full as possible and placed at different storage T_a_ (T_r_ approximately +20°C, refrigerator +4°C, freezers –20°C and –80°C and liquid nitrogen –196°C) yielding a total of 5 vials for each animal/T_a_. All vials were protected from light exposure.

### Fatty acid analyses

At 0, 1, 2, 3, 7, 28, 84 and 168 days, a small part of each sample was extracted for FA analysis. The subsamples were obtained by first removing the surface of a sample to gain tissue only from the inner part that would have been unaffected by exposure to air and light. The remaining sample was sealed in its storage vial after replacing air with nitrogen gas, and the procedure was repeated at each time point. FA from tissue total lipids were analyzed according to a procedure described previously [3]. Briefly, the samples were transmethylated by heating with 1% H_2_SO_4_ in methanol under nitrogen atmosphere, the FA methyl esters were extracted with hexane and analyzed by a gas–liquid chromatograph (6890N; Agilent Technologies Inc., Santa Clara, CA, USA) and the chromatograms were integrated manually with the Agilent ChemStation software (Agilent Technologies Inc.).

### Statistical analyses

The results of each sample at a particular T_a_ were compared to the initial FA values of the same animal by subtracting the mol-% of a sample from the initial value. The resulting differences were used for the mixed model analysis to evaluate the changes in FA proportions and the interaction time × T_a_ (IBM SPSS *v*21.0 software, IBM, Armonk, NY, USA). Due to multiple comparisons, the Bonferroni correction was used to control for false positives. The results are presented as the mean ± SE. To analyze the FAS as a whole, we also performed discriminant analysis to see how the FAS differed based on species, storage time and T_a_ and to see if the overall FAS would allow the correct classification of the samples into species and storage conditions.

## Results

As expected, the different species had clearly distinct FAS (S1 Table). The omnivorous rat represents a FAS with a higher proportion of n-6 PUFA than the other species, the piscivorous mink has the highest MUFA sum and the aquatic rainbow trout has high levels of long-chain n-3 PUFA.

According to the mixed model analysis, there were no statistically significant, consistent changes in the proportions of most major FA (>1 mol-%) during storage. For the rat samples, the FA with significant time × T_a_ interactions during the study were 15:0, 19:0*i*, 18:2*c*9*t*11, 18:4n-3, 20:1n-5, 20:2n-9, 22:1n-11, 22:0, 23:0 and 24:0. Of these, 18:2*c*9*t*11 (at all storage T_a_), 15:0 and 22:0 (at above-freezing T_a_) increased in proportion, while decreases were observed for 18:4n-3, 20:1n-5, 20:2n-9, 23:0 (all storage T_a_) and 22:1n-11 (below-freezing T_a_). For 19:0*i* and 24:0, the changes were mostly inconsistent fluctuations. For the mink, there were significant time × T_a_ interactions for 16:0, 18:2*c*9*t*11, 20:4n-3 and 21:5n-3 (decreases), 18:2n-6, 19:1n-10, 19:1n-8 (increases), and for 16:1n-5, 18:2n-4, 18:3n-3 and 22:5n-6 (inconsistent). Regarding the rainbow trout samples, significant time × T_a_ interactions were observed in 15:0 (increase at above-freezing and decrease at below-freezing T_a_), 22:0, 22:1n-9 (decreases) and 24:0 (slight increase).

**Fig 1:**
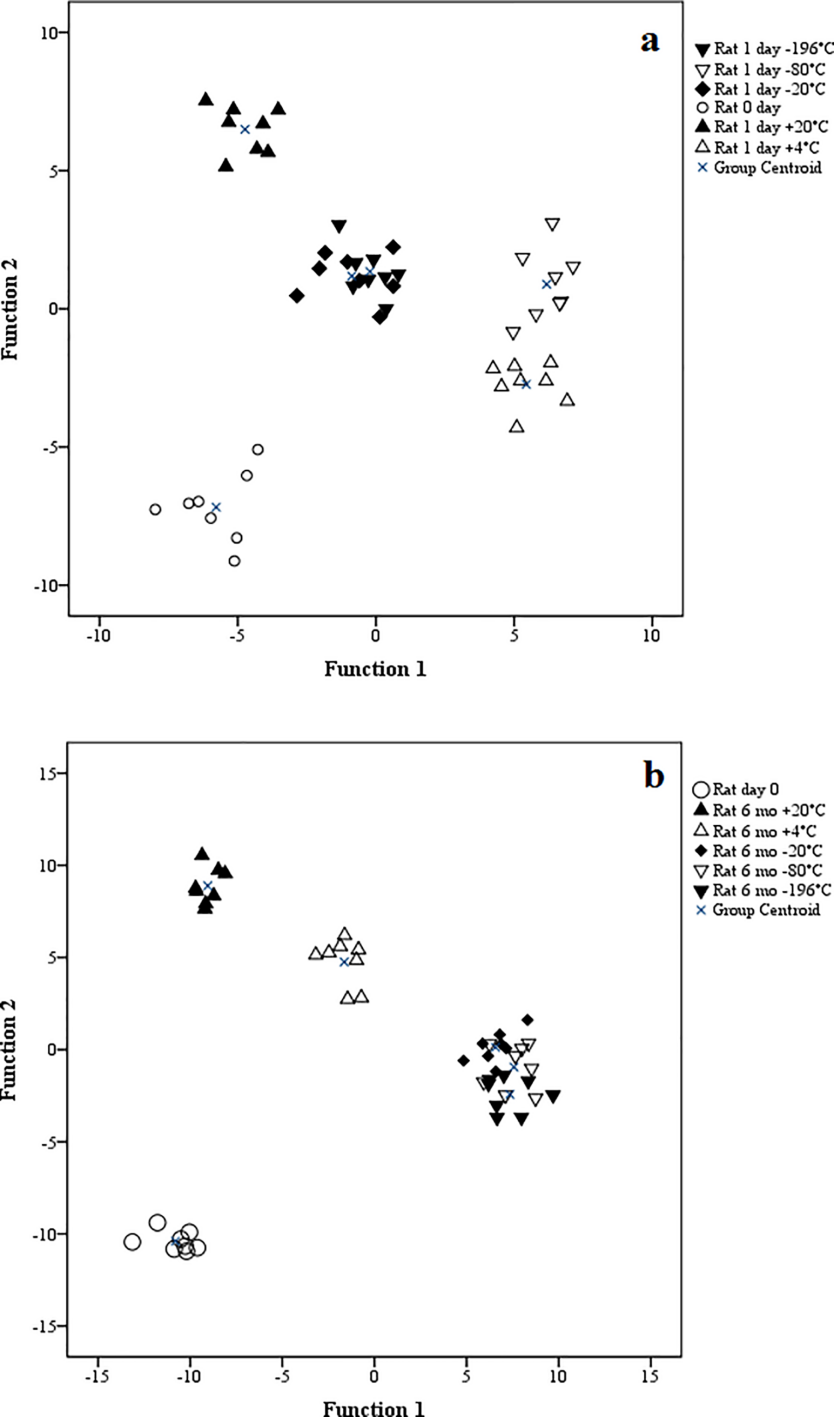
Discriminant analysis of rat fatty acids at sampling and after 1 day (a) or 6 months (b) at different storage temperatures.

**Fig 2:**
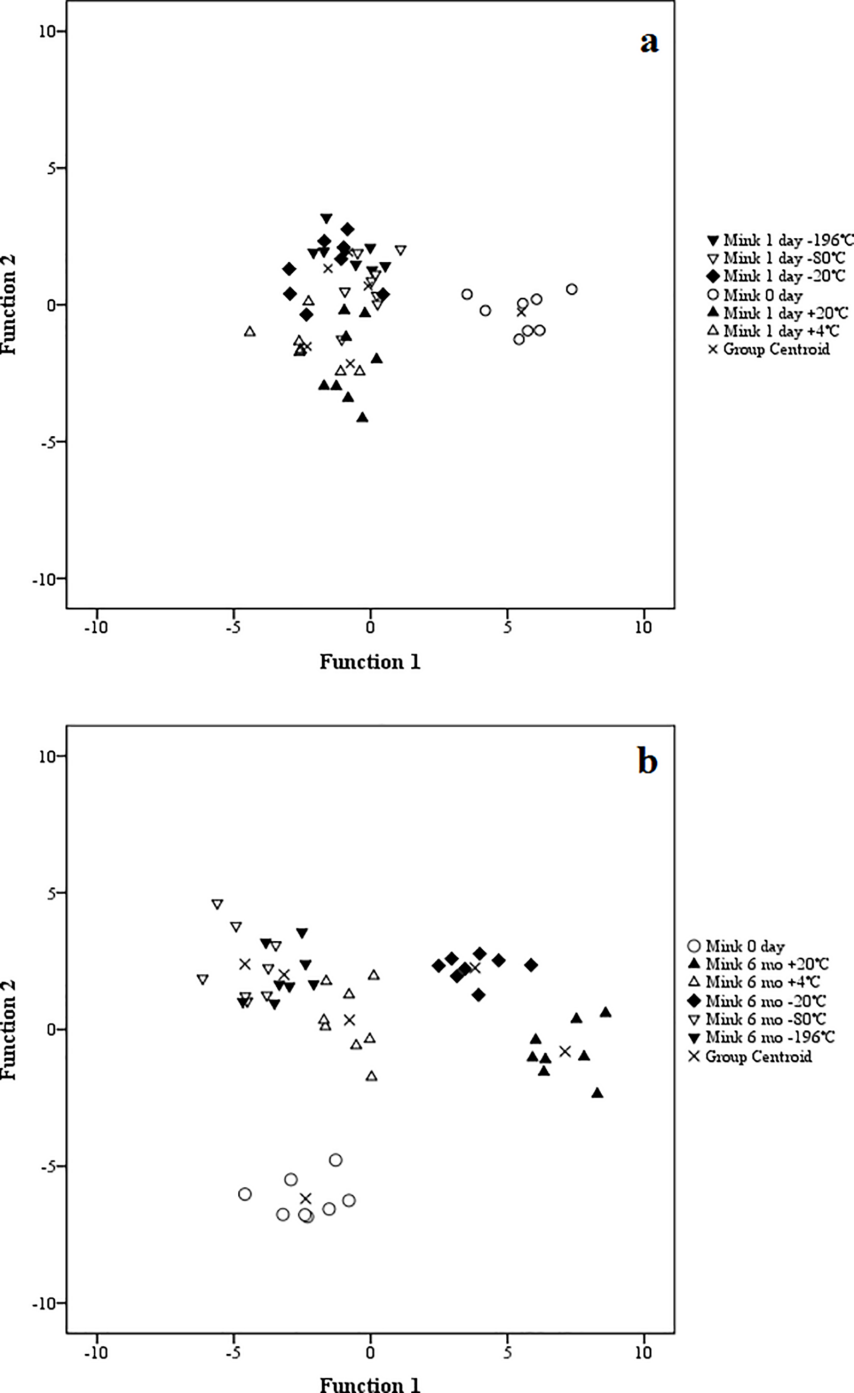
Discriminant analysis of American mink fatty acids at sampling and after 1 day (a) or 6 months (b) at different storage temperatures.

**Fig 3:**
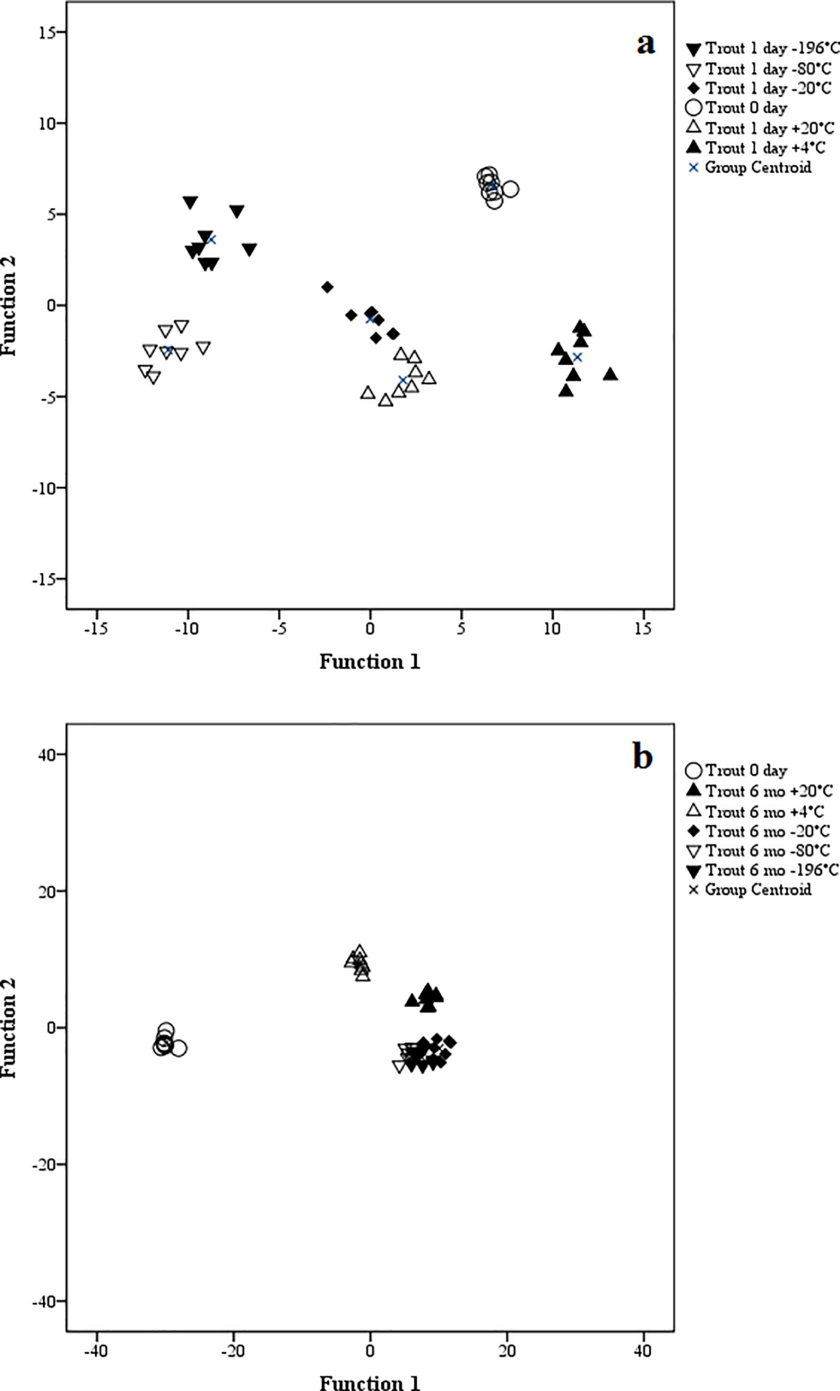
Discriminant analysis of rainbow trout fatty acids at sampling and after 1 day (a) or 6 months (b) at different storage temperatures.

In the discriminant analysis, all three species were clearly classified separate from the day of sampling until 6 months of storage regardless of storage T_a_ (S1 Fig). The FA in the structure matrix of the analysis with the highest correlations with the discriminant functions were 22:6n-3, 20:3n-3, 24:0, 17:0*ai* and 14:1n-9 (1 day) and 20:4n-3, 20:1n-5, 22:0, 18:2n-4 and 22:6n-3 (6 months). The discriminant analysis was also performed separately for each species by comparing the FAS from the fresh samples to the FA profile results after 1 day and 6 months of storage at different T_a_. Regardless of species, all stored samples at all T_a_ were clearly separated from the initial measurements as early as 1 day after the sampling (Figs 1A–3A) and after 6 months the difference remained evident (Figs 1B–3B). In the rat samples, the FA that separated the storage fats the most were 18:1n-7, 14:1n-9, 15:0, 18:4n-3 and 24:0 (1 day) and 24:0, 20:4n-3, 16:0*i*, 18:4n-3 and 15:0 (6 months). For the mink, the separating FA were 16:1n-9, 18:1n-9, 18:1n-7, 18:2n-4 and 20:1n-7 (1 day) and 18:2n-6, 16:1n-9, 16:0*i*, 20:3n-6 and 17:0*i* (6 months), and for the rainbow trout 17:0*ai*, 17:0*i*, 20:4n-3, 15:0 and 22:0 (1 day) and 20:1n-5, 22:6n-3, 20:4n-6, 15:0 and 15:0*i* (6 months). The analysis classified 100% of the samples correctly according to species at both time points. For the individual species at different T_a_, the analysis also classified the cases mostly correctly. At 6 months, one mink individual at –80°C was classified with the –196°C samples.

## Discussion

The results of the present study clearly showed, how the FAS remained unexpectedly stable for 6 months despite the very different storage T_a_. Generally, our data were able to confirm the previous observations by Matthan et al. [5] and Hodson et al. [6], who conducted analyses on human serum/plasma/erythrocyte FA profiles after long-term storage at –80°C, and the results of Lind et al. [10,11] with stable seal FAS even at +2°C up to 6 months, and fish FAS at –25°C over decades. Compared to the literature, the present study was more comprehensive with three sampled vertebrate species, several storage T_a_ and frequent re-analyses during the study period including one analysis immediately upon sampling. The rat represented a more n-6 PUFA-based FAS, the mink was the MUFA model and the rainbow trout was an example of the long-chain n-3 PUFA model. In addition, the discriminant analysis and the frequent sampling at various time points allowed us to discern when the general FA profiles would have started to diverge from the moment of sampling, if ever. Thus, we could assess the effects of i) species, ii) time and iii) T_a_ separately.

Regarding i) the species-specific differences, we could demonstrate with the discriminant analysis that fats from the examined species were classified clearly separate even after the 6-month storage at T_r_. The differences between the species, and not between the storage times or T_a_, clearly dominated the output, and the different FAS with emphasis on either MUFA or n-3/n-6 PUFA were maintained without any visible convergence of the species. Thus, we can conclude that the general characteristics of a species’ FAS remain intact despite long-term storage, even at T_r_.

When the effect of ii) time was considered, unexpectedly, the general differences between the FAS arose as early as 1 day after the initial sampling with the discriminant analysis showing clear classification of all stored fats apart from the initial FAS. Mostly in concert with our results and emphasizing the effect of time over T_a_, Deslypere et al. [15] examined n-3 PUFA in human lipid samples and noticed minor increases in the proportions of, *e*.*g*., 20:5n-3 and 22:6n-3 after 3 months regardless of storage T_a_, while 18:3n-3 decreased at T_r_ after 7 months. Some previous studies [5,6] did not always analyze the samples immediately after sampling but only after several months. As our results indicated that small differences can arise within 24 h, the initial analyses should be conducted on fresh samples when feasible and when very high precision is required.

To summarize, while there was an initial divergence in the overall FAS from the moment of sampling, further storage regardless of T_a_ did not significantly change the classification of the FA profiles any further. Compared to the temporal effect, the responses to iii) storage T_a_ could be less significant, as even the coldest T_a_ did not lead to the FAS to be classified together with the initial analysis results. Although the discriminant analysis at 6 months mostly classified the samples into either above- or below-freezing T_a_ (except of two of the mink groups), the general effect of all samples diverging from the initial analysis was more pronounced. While the analysis showed an overall effect of T_a_ on the FAS, the changes seemed not to be great enough to cause a significant risk of error in the interpretation of FAS. It is possible that all FA deteriorated in our samples at a similar rate meaning that the relative abundance of FA remained constant, even though the absolute lipid content probably declined over time [8]. As the focus is on relative FA proportions, this does not undermine the usefulness of stored samples for research purposes.

The FA with significant time × T_a_ interactions were mostly FA that are present in fat tissue in small or very small proportions. Some notable exceptions to this were the decrease in 16:0 and the increase in 18:2n-6 in the mink samples and, even in these cases, the changes from the original values were small albeit statistically significant in the mixed model analysis. Had the changes in lipid composition been caused by autoxidation, we would have expected to see consistently decreasing proportions of PUFA compared to SFA, especially those with a higher number of double bonds [13]. In contrast, we speculate that lipid autolysis would treat structurally different FA less selectively than generally assumed, and suppose that the process would be initiated by the post-mortem lack of ATP, pH change and release of Ca^2+^ followed by the enhanced enzymatic hydrolysis of lipids and partial degradation of the freed FA [16]. During the 6-months storage in warm conditions, there were some indications of probable autoxidation especially in the rainbow trout samples with slightly decreased proportions of 22:6n-3 (0 d: 6.7 ± 0.07 mol-%; 6 mo at +4°C: 6.3 ± 0.09 mol-%; 6 mo at +20°C: 6.2 ± 0.23 mol-%). While the good preservation of this PUFA could initially seem surprising, it fits very well with previous data. Lind et al. [11] examined the FAS of seal blubber without detecting significant changes when the samples were stored up to 6 months at +2°C and still only a few changes in two individuals (decreases in 20:4n-6 and 22:6n-3 and increases in 18:2n-6, 20:3n-3, 20:4n-3 and 22:4n-3) after 4–6 years at –25°C. The latter changes were suggested to be caused by differences in the collection of the samples. Similar to our observations, the decomposition of samples at >0°C was clearly observable, and the blubber showed degradation into liquid and solid fractions without major effects on the FAS. We also noticed the liquefaction of preserved samples at +20°C, but the FAS at this T_a_ were still maintained relatively intact. In selected n-3 PUFA in human lipid samples, good recovery was also noticed in the FA proportions even after 5.6 years at +20°C [17].

Why the relative abundance of FA would be so unaffected by long-term storage even at T_r_ is intriguing. Apparently, analyzing subsamples from the deeper tissue layers, the least exposed to air, could be a factor to prevent autoxidative effects from being visible. Also in the case of samples showing decomposition and/or liquefaction, the subsamples for analysis were not taken from the surfaces of the vials. Microbiological activity could have affected the samples at above-freezing T_a_, but little indication of this was observed in the relative FA abundances. Finally, the general change in the FAS, as observed in the discriminant analysis, indicated that enzymatic autolysis may have caused this initial divergence, which did not widen after the reaction had run its course. The fact that the compositional change during day 1 was visible at all T_a_, and not just in the frozen samples, suggests that ice formation and subsequent organelle breaking were not required to release the autolytic enzymes. Instead, this phenomenon of lysosomal degradation would have been induced by post-mortem depletion of ATP [16], which halts the proton pumps of lysosomal membranes and at the same time induces glycolytic lactate production. Consequently, the cytosolic pH decreases and the acidic lipases leaking from lysosomes are activated. The liberated free FA are surface-active and seek lipid–water interfaces, where they are more prone to further degradation than, *e*.*g*., the FA bound to neutral lipids and, thus, protected inside the lipid droplets. This lipolytic process probably continued at a low rate for some time even after the sample temperature decreased [18]. Previously, Lind et al. [11] suggested that the good preservation of FAS during storage could be related to the presence of an antioxidant, vitamin E. Together with other antioxidative substances it may explain how oxidation was delayed but not totally prevented in the rainbow trout lipid samples.

## Conclusions

Contrary to our hypothesis i), FAS were very well preserved for 6 months even at T_r_ when adequately protected from exposure to air and light. The main effects of time were visible as early as 1 day after the initial measurement but did not greatly increase over longer periods. ii) FA structure did not clearly affect the preservation as only relatively few FA mostly with minor proportions showed significant and consistent changes during storage. iii) The FAS at different T_a_ showed grouping into above- and sub-freezing T_a_, but the differences do not invalidate the analysis of older samples regardless of storage T_a_. In addition, the general species-specific FA profiles remained intact and clearly separate throughout storage.

## Supporting information

S1 Fig Discriminant analysis of the fatty acid proportions of the studied species at sampling and after 6 months of storage. The fatty acid signatures separate the species very clearly despite the long-term storage even at +20°C.
(TIF)

S1 Table Proportions (mol-%) of selected fatty acids (FA) and their sums in the studied species at sampling and after 6 months of storage at different temperatures (mean ± SE, n = 8 for each species and sampling/temperature). (DOCX)

S2 Table Raw dataset analyzed in the present study. (SAV)

## Abbreviations

ai: anteiso
ATP: adenosine triphosphate
c: cis
FA: fatty acid
FAS: fatty acid signature
i: iso
MUFA: monounsaturated fatty acid
PUFA: polyunsaturated fatty acid
SFA: saturated fatty acid, *t* trans
T_a_: ambient temperature
TAG: triacylglycerol
T_r_: room temperature

## References

[1] Käkelä A, Crane J, Votier SC, Furness RW, Käkelä R. Fatty acid signatures as indicators of diet in great skuas *Stercorarius skua*, Shetland. Mar Ecol Prog Ser. 2006;319: 297–310.

[2] Käkelä A, Furness RW, Kelly A, Strandberg U, Waldron S, Käkelä R. Fatty acid signatures and stable isotopes as dietary indicators in North Sea seabirds. Mar Ecol Prog Ser. 2007; 342:291–301.

[3] Mustonen A-M, Asikainen J, Aho J, Nieminen P. Selective seasonal fatty acid accumulation and mobilization in the wild raccoon dog (*Nyctereutes procyonoides*). Lipids. 2007; 42:1155–1167. 10.1007/s11745-007-3118-5 17926077

[4] Galicia MP, Thiemann GW, Dyck MG, Ferguson SH, Higdon JW. Dietary habits of polar bears in Foxe Basin, Canada: possible evidence of a trophic regime shift mediated by a new top predator. Ecol Evol. 2016;6: 6005–6018. 10.1002/ece3.2173 27547372 PMC4983609

[5] Matthan NR, Ip B, Resteghini N, Ausman LM, Lichtenstein AH. Long-term fatty acid stability in human serum cholesteryl ester, triclygeride, and phospholipid fractions. J Lipid Res. 2010;51: 2826–2832. 10.1194/jlr.D007534 20448292 PMC2918465

[6] Hodson L, Skeaff CM, Wallace AJ, Arribas GLB. Stability of plasma and erythrocyte fatty acid composition during cold storage. Clin Chim Acta. 2002;321: 63–67. 12031594 10.1016/s0009-8981(02)00100-6

[7] Beynen AC, Katan MB. Rapid sampling and long-term storage of subcutaneous adipose-tissue biopsies for determination of fatty acid composition. Am J Clin Nutr. 1985;42: 317–322. 10.1093/ajcn/42.2.317 4025201

[8] Balev D, Ivanov G, Dragoev S, Nikolov H. Effect of vacuum-packaging on the changes of Russian sturgeon muscle lipids during frozen storage. Eur J Lipid Sci Technol. 2011;113: 1385–1394.

[9] Vali Hosseini S, Abedian-Kenari A, Rezaei M, Nazari RM, Feás X, Rabbani M. Influence of the in vivo addition of alpha-tocopheryl acetate with three lipid sources on the lipid oxidation and fatty acid composition of Beluga sturgeon, *Huso huso*, during frozen storage. Food Chem. 2010;118: 341–348.

[10] Lind Y, Huovila T, Käkelä R. A retrospective study of fatty acid composition in Baltic herring (*Clupea harengus membras*) caught at three locations in the Baltic Sea (1973–2009). ICES J Mar Sci. 2018;75: 330–339.

[11] Lind Y, Bäcklin B-M, Lundström K, Budge SM, Walton M, Karlsson O. Stability of fatty acid composition in seal blubber during long-term storage. Mar Ecol Prog Ser. 2012;461: 283–291.

[12] Pinheiro J. Decay process of a cadaver In: Schmitt A, Cunha E, Pinheiro J, editors. Forensic anthropology and medicine. Complementary sciences from recovery to cause of death. Totowa: Humana Press; 2006 pp. 85–116.

[13] deMan JM. Chemical and physical properties of fatty acids In: Chow CK, editor. Fatty acids in foods *and their* health implications, 3rd edition Boca Raton: CRC Press; 2008 pp. 17–45.

[14] Paczkowski S, Schütz S. Post-mortem volatiles of vertebrate tissue. Appl Microbiol Biotechnol. 2011;91: 917–935. 10.1007/s00253-011-3417-x 21720824 PMC3145088

[15] Deslypere J-P, van de Bovenkamp P, Harryvan JL, Katan MB. Stability of n-3 fatty acids in human fat tissue aspirates during storage. Am J Clin Nutr. 1993;57: 884–888. 10.1093/ajcn/57.6.884 8503357

[16] Huss HH. Fresh fish—Quality and quality changes Rome: FAO; 1988.

[17] Katan MB, Harryvan JL, van de Bovenkamp P. N-3 fatty acids in human fat tissue aspirates are stable for up to 6 y. Eur J Clin Nutr. 2003;57: 816–818. 10.1038/sj.ejcn.1601614 12821880

[18] Geromel EJ, Montgomery MW. Lipase release from lysosomes of rainbow trout (*Salmo gairdneri*) muscle subjected to low temperatures. J Food Sci. 1980;45: 412–415.

