# Supplementary figures and images for: Preservation of fatty acid signatures in three vertebrate species after six months of storage at various temperatures
Source: PLoS One. 2018 Sep 17;13(9):e0204207. doi: 10.1371/journal.pone.0204207 (PMC6141075; doi:10.1371/journal.pone.0204207)

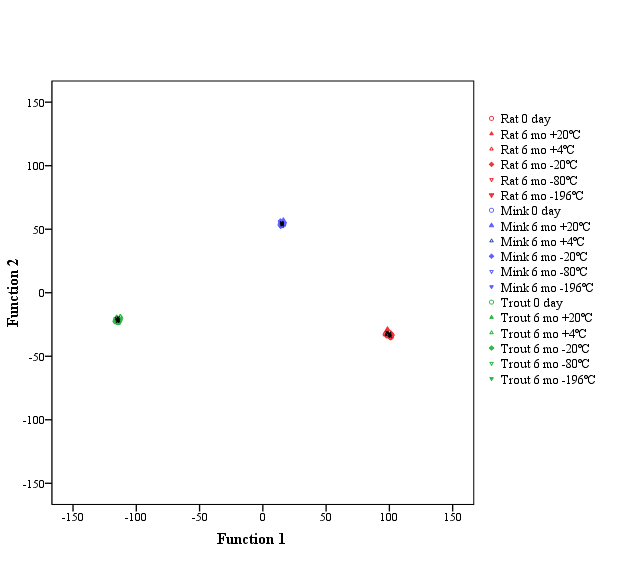

Supplement: S1 Fig — The fatty acid signatures separate the species very clearly despite the long-term storage even at +20°C. (TIF) [file pone.0204207.s001.tif]
