# Supplementary material for: Preservation of fatty acid signatures in three vertebrate species after six months of storage at various temperatures
Source: PLoS One. 2018 Sep 17;13(9):e0204207. doi: 10.1371/journal.pone.0204207 (PMC6141075; doi:10.1371/journal.pone.0204207)
Supplement: S1 Table — (DOCX) [file pone.0204207.s002.docx]

**S1 Table**. Proportions (mol-%) of selected fatty acids (FA) and their sums in the studied species at sampling and after 6 months of storage at different temperatures (mean ± SE, n = 8 for each species and sampling/temperature).

|  | Rat | | | | | | American mink | | | | | | Rainbow trout | | | | | |
| --- | --- | --- | --- | --- | --- | --- | --- | --- | --- | --- | --- | --- | --- | --- | --- | --- | --- | --- |
| FA | Sampling | –196ºC | –80ºC | –20ºC | +4ºC | +20ºC | Sampling | –196ºC | –80ºC | –20ºC | +4ºC | +20ºC | Sampling | –196ºC | –80ºC | –20ºC | +4ºC | +20ºC |
| 14:0 | 1.254 ± 0.084 | 1.082 ± 0.046 | 1.176 ± 0.057 | 1.236 ± 0.080 | 1.177 ± 0.052 | 1.212 ± 0.064 | 4.021 ± 0.248 | 3.740 ± 0.131 | 3.561 ± 0.191 | 3.718 ± 0.110 | 3.861 ± 0.229 | 3.928 ± 0.156 | 5.605 ± 0.060 | 5.541 ± 0.067 | 5.509 ± 0.036 | 5.503 ± 0.051 | 5.706 ± 0.094 | 5.744 ± 0.083 |
| 14:1n-9 | 0.007 ± <0.001 | 0.007 ± 0.001 | 0.006 ± <0.001 | 0.007 ± 0.001 | 0.008 ± <0.001 | 0.009 ± 0.001 | 0.020 ± 0.001 | 0.020 ± 0.001 | 0.019 ± 0.001 | 0.021 ± 0.001 | 0.021 ± 0.001 | 0.021 ± 0.001 | 0.039 ± 0.001 | 0.042 ± 0.001 | 0.042 ± 0.001 | 0.043 ± 0.001 | 0.043 ± 0.001 | 0.044 ± 0.001 |
| 14:1n-7 | 0.018 ± 0.001 | 0.013 ± 0.001 | 0.013 ± 0.001 | 0.015 ± 0.002 | 0.012 ± 0.001 | 0.014 ± 0.001 | 0.069 ± 0.006 | 0.067 ± 0.005 | 0.067 ± 0.005 | 0.072 ± 0.005 | 0.071 ± 0.005 | 0.070 ± 0.006 | 0.039 ± 0.002 | 0.040 ± 0.001 | 0.039 ± 0.001 | 0.041 ± 0.001 | 0.044 ± 0.001 | 0.043 ± 0.001 |
| 14:1n-5 | 0.038 ± 0.003 | 0.023 ± 0.003 | 0.027 ± 0.003 | 0.038 ± 0.009 | 0.025 ± 0.003 | 0.032 ± 0.004 | 0.433 ± 0.060 | 0.386 ± 0.051 | 0.337 ± 0.041 | 0.414 ± 0.030 | 0.443 ± 0.085 | 0.444 ± 0.076 | 0.058 ± 0.001 | 0.064 ± 0.001 | 0.062 ± 0.001 | 0.064 ± 0.001 | 0.064 ± 0.002 | 0.066 ± 0.001 |
| 15:0*i* | 0.009 ± 0.001 | 0.008 ± 0.001 | 0.008 ± 0.001 | 0.008 ± 0.001 | 0.008 ± 0.001 | 0.009 ± 0.001 | 0.104 ± 0.004 | 0.104 ± 0.004 | 0.104 ± 0.004 | 0.106 ± 0.003 | 0.103 ± 0.002 | 0.107 ± 0.003 | 0.183 ± 0.002 | 0.187 ± 0.002 | 0.186 ± 0.001 | 0.189 ± 0.002 | 0.191 ± 0.002 | 0.196 ± 0.002 |
| 15:0*ai* | 0.014 ± 0.001 | 0.014 ± 0.002 | 0.013 ± 0.001 | 0.014 ± 0.001 | 0.013 ± 0.001 | 0.013 ± 0.001 | 0.052 ± 0.002 | 0.054 ± 0.001 | 0.055 ± 0.001 | 0.056 ± 0.001 | 0.053 ± 0.001 | 0.056 ± 0.001 | 0.059 ± 0.001 | 0.061 ± 0.001 | 0.060 ± 0.001 | 0.062 ± 0.001 | 0.062 ± 0.001 | 0.065 ± 0.001 |
| 15:0 | 0.240 ± 0.006 | 0.251 ± 0.008 | 0.247 ± 0.006 | 0.250 ± 0.007 | 0.284 ± 0.011 | 0.260 ± 0.005 | 0.268 ± 0.010 | 0.261 ± 0.003 | 0.262 ± 0.002 | 0.254 ± 0.001 | 0.259 ± 0.003 | 0.262 ± 0.003 | 0.382 ± 0.004 | 0.373 ± 0.005 | 0.374 ± 0.003 | 0.375 ± 0.005 | 0.395 ± 0.006 | 0.393 ± 0.008 |
| 15:1n-6 | 0.015 ± 0.001 | 0.015 ± <0.001 | 0.015 ± 0.001 | 0.015 ± 0.001 | 0.016 ± 0.001 | 0.015 ± 0.001 | 0.023 ± 0.002 | 0.021 ± 0.002 | 0.020 ± 0.001 | 0.024 ± 0.001 | 0.023 ± 0.002 | 0.024 ± 0.003 | 0.010 ± 0.001 | 0.010 ± <0.001 | 0.010 ± <0.001 | 0.010 ± <0.001 | 0.010 ± 0.001 | 0.010 ± <0.001 |
| 16:0*i* | 0.044 ± 0.001 | 0.042 ± 0.001 | 0.042 ± 0.001 | 0.043 ± 0.001 | 0.044 ± 0.001 | 0.044 ± 0.001 | 0.051 ± 0.001 | 0.049 ± 0.001 | 0.048 ± 0.001 | 0.048 ± 0.001 | 0.049 ± 0.001 | 0.051 ± 0.001 | 0.070 ± 0.001 | 0.070 ± 0.001 | 0.070 ± 0.001 | 0.071 ± 0.001 | 0.072 ± 0.001 | 0.073 ± 0.001 |
| 16:0 | 18.970 ± 0.209 | 18.321 ± 0.230 | 18.684 ± 0.275 | 19.085 ± 0.382 | 18.987 ± 0.245 | 19.250 ± 0.339 | 18.786 ± 0.906 | 18.028 ± 0.443 | 18.009 ± 0.355 | 16.285 ± 0.541 | 17.844 ± 0.404 | 18.369 ± 0.454 | 13.963 ± 0.105 | 13.790 ± 0.097 | 13.810 ± 0.109 | 13.705 ± 0.123 | 14.162 ± 0.118 | 14.148 ± 0.215 |
| 16:1n-9 | 0.470 ± 0.007 | 0.472 ± 0.004 | 0.457 ± 0.005 | 0.468 ± 0.008 | 0.464 ± 0.005 | 0.463 ± 0.006 | 0.477 ± 0.014 | 0.505 ± 0.014 | 0.516 ± 0.009 | 0.520 ± 0.012 | 0.503 ± 0.012 | 0.496 ± 0.012 | 0.325 ± 0.012 | 0.285 ± 0.008 | 0.282 ± 0.012 | 0.262 ± 0.009 | 0.281 ± 0.009 | 0.258 ± 0.014 |
| 16:1n-7 | 1.907 ± 0.188 | 1.233 ± 0.133 | 1.352 ± 0.142 | 1.723 ± 0.291 | 1.222 ± 0.117 | 1.502 ± 0.166 | 10.684 ± 1.058 | 9.902 ± 0.900 | 9.238 ± 0.685 | 10.535 ± 0.533 | 10.453 ± 0.907 | 10.730 ± 1.104 | 6.370 ± 0.075 | 6.402 ± 0.064 | 6.363 ± 0.064 | 6.413 ± 0.075 | 6.477 ± 0.085 | 6.395 ± 0.069 |
| 16:1n-5 | 0.068 ± 0.003 | 0.069 ± 0.003 | 0.069 ± 0.003 | 0.070 ± 0.003 | 0.070 ± 0.003 | 0.070 ± 0.003 | 0.147 ± 0.003 | 0.150 ± 0.001 | 0.152 ± 0.001 | 0.155 ± 0.001 | 0.151 ± 0.001 | 0.149 ± 0.002 | 0.163 ± 0.002 | 0.166 ± 0.001 | 0.165 ± 0.002 | 0.167 ± 0.002 | 0.168 ± 0.002 | 0.171 ± 0.002 |
| 17:0*i* | 0.141 ± 0.003 | 0.145 ± 0.003 | 0.141 ± 0.003 | 0.141 ± 0.003 | 0.142 ± 0.003 | 0.140 ± 0.002 | 0.102 ± 0.004 | 0.096 ± 0.002 | 0.095 ± 0.002 | 0.092 ± 0.002 | 0.096 ± 0.002 | 0.097 ± 0.003 | 0.141 ± 0.004 | 0.141 ± 0.002 | 0.142 ± 0.002 | 0.143 ± 0.002 | 0.144 ± 0.002 | 0.142 ± 0.002 |
| 17:0*ai* | 0.215 ± 0.006 | 0.235 ± 0.006 | 0.233 ± 0.006 | 0.229 ± 0.006 | 0.241 ± 0.005 | 0.236 ± 0.006 | 0.128 ± 0.003 | 0.133 ± 0.002 | 0.136 ± 0.002 | 0.135 ± 0.003 | 0.134 ± 0.002 | 0.134 ± 0.002 | 0.036 ± 0.003 | 0.055 ± 0.002 | 0.057 ± 0.003 | 0.062 ± 0.002 | 0.053 ± 0.002 | 0.042 ± 0.002 |
| 17:0 | 0.125 ± 0.003 | 0.112 ± 0.002 | 0.115 ± 0.003 | 0.120 ± 0.004 | 0.115 ± 0.002 | 0.118 ± 0.003 | 0.214 ± 0.016 | 0.216 ± 0.012 | 0.225 ± 0.009 | 0.203 ± 0.005 | 0.209 ± 0.012 | 0.210 ± 0.014 | 0.196 ± 0.002 | 0.195 ± 0.003 | 0.196 ± 0.002 | 0.199 ± 0.003 | 0.204 ± 0.002 | 0.204 ± 0.003 |
| 17:1n-8 | 0.051 ± 0.002 | 0.057 ± 0.003 | 0.055 ± 0.003 | 0.055 ± 0.003 | 0.057 ± 0.003 | 0.056 ± 0.003 | 0.304 ± 0.014 | 0.290 ± 0.013 | 0.278 ± 0.007 | 0.293 ± 0.005 | 0.293 ± 0.008 | 0.295 ± 0.008 | 0.564 ± 0.009 | 0.567 ± 0.006 | 0.560 ± 0.007 | 0.566 ± 0.003 | 0.567 ± 0.010 | 0.573 ± 0.007 |
| 18:0*i* | 0.013 ± 0.001 | 0.014 ± <0.001 | 0.014 ± <0.001 | 0.014 ± <0.001 | 0.014 ± <0.001 | 0.014 ± 0.001 | 0.044 ± 0.002 | 0.043 ± 0.001 | 0.043 ± 0.001 | 0.042 ± 0.001 | 0.045 ± 0.002 | 0.045 ± 0.002 | 0.130 ± 0.002 | 0.131 ± 0.001 | 0.132 ± 0.001 | 0.133 ± 0.001 | 0.134 ± 0.001 | 0.138 ± 0.002 |
| 18:0 | 2.853 ± 0.095 | 3.173 ± 0.074 | 3.194 ± 0.106 | 3.083 ± 0.078 | 3.257 ± 0.072 | 3.224 ± 0.107 | 3.329 ± 0.417 | 3.437 ± 0.343 | 3.645 ± 0.244 | 3.169 ± 0.166 | 3.243 ± 0.282 | 3.274 ± 0.350 | 2.498 ± 0.044 | 2.487 ± 0.047 | 2.492 ± 0.049 | 2.485 ± 0.051 | 2.557 ± 0.062 | 2.558 ± 0.041 |
| 18:1n-9 | 22.609 ± 0.166 | 22.686 ± 0.067 | 22.528 ± 0.089 | 22.409 ± 0.152 | 22.659 ± 0.155 | 22.580 ± 0.087 | 33.626 ± 0.819 | 34.777 ± 0.470 | 35.405 ± 0.587 | 35.679 ± 0.453 | 34.568 ± 0.677 | 34.311 ± 0.548 | 23.743 ± 0.203 | 23.882 ± 0.212 | 23.832 ± 0.195 | 23.983 ± 0.154 | 24.160 ± 0.191 | 24.144 ± 0.262 |
| 18:1n-7 | 2.269 ± 0.025 | 2.333 ± 0.038 | 2.274 ± 0.018 | 2.298 ± 0.028 | 2.317 ± 0.034 | 2.327 ± 0.039 | 2.936 ± 0.074 | 3.015 ± 0.058 | 3.072 ± 0.048 | 3.141 ± 0.043 | 3.024 ± 0.071 | 3.000 ± 0.061 | 2.623 ± 0.015 | 2.539 ± 0.033 | 2.548 ± 0.017 | 2.530 ± 0.026 | 2.632 ± 0.017 | 2.633 ± 0.097 |
| 18:1n-5 | 0.048 ± 0.001 | 0.049 ± 0.001 | 0.049 ± 0.001 | 0.050 ± 0.001 | 0.049 ± 0.001 | 0.048 ± 0.001 | 0.103 ± 0.004 | 0.101 ± 0.001 | 0.101 ± 0.002 | 0.105 ± 0.001 | 0.101 ± 0.001 | 0.103 ± 0.002 | 0.238 ± 0.002 | 0.233 ± 0.004 | 0.237 ± 0.003 | 0.236 ± 0.003 | 0.238 ± 0.004 | 0.239 ± 0.002 |
| 18:2n-6 | 42.978 ± 0.336 | 44.512 ± 0.306 | 44.185 ± 0.344 | 43.495 ± 0.610 | 43.635 ± 0.255 | 43.065 ± 0.390 | 14.928 ± 0.340 | 15.512 ± 0.187 | 15.656 ± 0.211 | 15.775 ± 0.202 | 15.341 ± 0.213 | 15.039 ± 0.235 | 8.967 ± 0.112 | 8.977 ± 0.124 | 8.952 ± 0.170 | 9.031 ± 0.123 | 8.968 ± 0.121 | 8.970 ± 0.106 |
| 18:3n-3 | 2.626 ± 0.056 | 2.616 ± 0.050 | 2.684 ± 0.039 | 2.684 ± 0.042 | 2.563 ± 0.058 | 2.558 ± 0.046 | 2.171 ± 0.066 | 2.203 ± 0.058 | 2.159 ± 0.046 | 2.223 ± 0.049 | 2.205 ± 0.050 | 2.127 ± 0.048 | 2.773 ± 0.056 | 2.797 ± 0.073 | 2.763 ± 0.071 | 2.792 ± 0.053 | 2.734 ± 0.070 | 2.736 ± 0.059 |
| 18:4n-3 | 0.009 ± 0.001 | 0.005 ± <0.001 | 0.005 ± <0.001 | 0.005 ± <0.001 | 0.005 ± <0.001 | 0.004 ± <0.001 | 0.406 ± 0.024 | 0.398 ± 0.025 | 0.385 ± 0.022 | 0.397 ± 0.023 | 0.396 ± 0.024 | 0.375 ± 0.022 | 1.194 ± 0.032 | 1.185 ± 0.027 | 1.181 ± 0.031 | 1.185 ± 0.033 | 1.138 ± 0.041 | 1.131 ± 0.039 |
| 20:0 | 0.063 ± 0.003 | 0.071 ± 0.004 | 0.069 ± 0.004 | 0.062 ± 0.004 | 0.073 ± 0.004 | 0.070 ± 0.004 | 0.096 ± 0.015 | 0.099 ± 0.013 | 0.105 ± 0.010 | 0.093 ± 0.006 | 0.093 ± 0.009 | 0.097 ± 0.010 | 0.156 ± 0.003 | 0.131 ± 0.005 | 0.127 ± 0.003 | 0.126 ± 0.005 | 0.147 ± 0.005 | 0.154 ± 0.003 |
| 20:1n-9 | 0.243 ± 0.007 | 0.241 ± 0.007 | 0.238 ± 0.007 | 0.227 ± 0.006 | 0.244 ± 0.006 | 0.249 ± 0.007 | 0.833 ± 0.060 | 0.889 ± 0.051 | 0.938 ± 0.050 | 0.915 ± 0.034 | 0.857 ± 0.040 | 0.869 ± 0.044 | 4.650 ± 0.064 | 4.716 ± 0.077 | 4.855 ± 0.082 | 4.805 ± 0.077 | 4.792 ± 0.131 | 4.777 ± 0.078 |
| 20:1n-7 | 0.225 ± 0.010 | 0.236 ± 0.012 | 0.231 ± 0.009 | 0.220 ± 0.011 | 0.233 ± 0.009 | 0.229 ± 0.009 | 0.102 ± 0.009 | 0.108 ± 0.008 | 0.116 ± 0.007 | 0.117 ± 0.005 | 0.106 ± 0.007 | 0.115 ± 0.006 | 0.225 ± 0.003 | 0.219 ± 0.003 | 0.222 ± 0.003 | 0.222 ± 0.003 | 0.223 ± 0.006 | 0.229 ± 0.003 |
| 20:2n-6 | 0.269 ± 0.015 | 0.233 ± 0.007 | 0.233 ± 0.008 | 0.243 ± 0.009 | 0.224 ± 0.004 | 0.235 ± 0.003 | 0.357 ± 0.013 | 0.340 ± 0.013 | 0.345 ± 0.012 | 0.350 ± 0.014 | 0.336 ± 0.009 | 0.339 ± 0.013 | 0.412 ± 0.007 | 0.409 ± 0.006 | 0.405 ± 0.006 | 0.412 ± 0.006 | 0.408 ± 0.007 | 0.412 ± 0.008 |
| 20:3n-6 | 0.092 ± 0.004 | 0.083 ± 0.005 | 0.079 ± 0.005 | 0.085 ± 0.007 | 0.073 ± 0.003 | 0.077 ± 0.004 | 0.107 ± 0.015 | 0.084 ± 0.005 | 0.082 ± 0.005 | 0.087 ± 0.004 | 0.088 ± 0.004 | 0.084 ± 0.005 | 0.186 ± 0.013 | 0.171 ± 0.012 | 0.170 ± 0.013 | 0.173 ± 0.012 | 0.167 ± 0.012 | 0.174 ± 0.013 |
| 20:4n-6 | 0.555 ± 0.021 | 0.604 ± 0.042 | 0.561 ± 0.027 | 0.634 ± 0.044 | 0.521 ± 0.022 | 0.543 ± 0.022 | 0.280 ± 0.018 | 0.268 ± 0.017 | 0.254 ± 0.013 | 0.265 ± 0.010 | 0.274 ± 0.017 | 0.264 ± 0.016 | 0.388 ± 0.005 | 0.400 ± 0.005 | 0.397 ± 0.003 | 0.398 ± 0.007 | 0.375 ± 0.003 | 0.379 ± 0.005 |
| 20:3n-3 | 0.026 ± 0.001 | 0.020 ± 0.001 | 0.021 ± 0.001 | 0.023 ± 0.002 | 0.021 ± 0.001 | 0.022 ± 0.001 | 0.180 ± 0.006 | 0.176 ± 0.008 | 0.170 ± 0.006 | 0.172 ± 0.006 | 0.172 ± 0.005 | 0.161 ± 0.005 | 0.181 ± 0.005 | 0.174 ± 0.005 | 0.170 ± 0.005 | 0.175 ± 0.005 | 0.168 ± 0.005 | 0.175 ± 0.006 |
| 20:4n-3 | 0.020 ± 0.003 | 0.014 ± 0.001 | 0.014 ± 0.001 | 0.013 ± 0.001 | 0.022 ± 0.001 | 0.023 ± 0.001 | 0.244 ± 0.016 | 0.236 ± 0.016 | 0.223 ± 0.013 | 0.235 ± 0.012 | 0.243 ± 0.016 | 0.227 ± 0.013 | 1.015 ± 0.026 | 1.032 ± 0.021 | 1.025 ± 0.019 | 1.034 ± 0.022 | 0.976 ± 0.019 | 1.002 ± 0.020 |
| 22:0 | 0.028 ± 0.004 | 0.018 ± 0.002 | 0.018 ± 0.002 | 0.015 ± 0.002 | 0.050 ± 0.004 | 0.049 ± 0.004 | 0.016 ± 0.003 | 0.016 ± 0.003 | 0.019 ± 0.003 | 0.016 ± 0.001 | 0.017 ± 0.002 | 0.016 ± 0.002 | 0.084 ± 0.005 | 0.065 ± 0.002 | 0.066 ± 0.002 | 0.067 ± 0.001 | 0.068 ± 0.003 | 0.067 ± 0.002 |
| 22:1n-11 | 0.065 ± 0.009 | 0.032 ± 0.003 | 0.033 ± 0.003 | 0.027 ± 0.002 | 0.081 ± 0.005 | 0.086 ± 0.005 | 0.088 ± 0.014 | 0.100 ± 0.010 | 0.110 ± 0.010 | 0.107 ± 0.007 | 0.092 ± 0.007 | 0.102 ± 0.008 | 3.901 ± 0.075 | 3.801 ± 0.085 | 3.947 ± 0.105 | 3.837 ± 0.077 | 3.855 ± 0.140 | 3.838 ± 0.116 |
| 22:5n-3 | 0.123 ± 0.004 | 0.103 ± 0.005 | 0.100 ± 0.008 | 0.113 ± 0.008 | 0.101 ± 0.005 | 0.098 ± 0.003 | 0.416 ± 0.024 | 0.382 ± 0.027 | 0.373 ± 0.021 | 0.390 ± 0.024 | 0.397 ± 0.028 | 0.370 ± 0.028 | 1.679 ± 0.041 | 1.745 ± 0.037 | 1.729 ± 0.037 | 1.756 ± 0.038 | 1.630 ± 0.027 | 1.633 ± 0.039 |
| 22:6n-3 | 0.086 ± 0.004 | 0.076 ± 0.004 | 0.076 ± 0.005 | 0.085 ± 0.005 | 0.070 ± 0.002 | 0.085 ± 0.003 | 1.736 ± 0.141 | 1.653 ± 0.146 | 1.572 ± 0.130 | 1.630 ± 0.141 | 1.699 ± 0.154 | 1.570 ± 0.147 | 6.713 ± 0.066 | 6.963 ± 0.084 | 6.990 ± 0.109 | 6.969 ± 0.086 | 6.321 ± 0.094 | 6.153 ± 0.233 |
| 24:0 | 0.006 ± 0.001 | 0.003 ± <0.001 | 0.003 ± <0.001 | 0.003 ± <0.001 | 0.005 ± 0.001 | 0.006 ± 0.001 | 0.020 ± 0.007 | 0.007 ± 0.001 | 0.009 ± 0.001 | 0.008 ± 0.001 | 0.007 ± 0.001 | 0.010 ± 0.001 | 0.014 ± 0.001 | 0.016 ± 0.001 | 0.017 ± 0.001 | 0.016 ± 0.001 | 0.018 ± 0.001 | 0.018 ± 0.002 |
| SFA | 24.152 ± 0.282 | 23.653 ± 0.198 | 24.122 ± 0.278 | 24.467 ± 0.398 | 24.561 ± 0.247 | 24.793 ± 0.360 | 27.523 ± 1.390 | 26.577 ± 0.715 | 26.603 ± 0.490 | 24.523 ± 0.520 | 26.312 ± 0.622 | 26.951 ± 0.683 | 23.679 ± 0.119 | 23.390 ± 0.131 | 23.383 ± 0.154 | 23.285 ± 0.188 | 24.074 ± 0.197 | 24.214 ± 0.413 |
| MUFA | 28.139 ± 0.254 | 27.546 ± 0.134 | 27.425 ± 0.082 | 27.697 ± 0.202 | 27.539 ± 0.170 | 27.763 ± 0.163 | 50.297 ± 1.227 | 50.858 ± 0.754 | 50.922 ± 0.575 | 52.642 ± 0.540 | 51.191 ± 0.605 | 51.226 ± 0.763 | 45.114 ± 0.185 | 44.994 ± 0.210 | 45.180 ± 0.155 | 45.158 ± 0.183 | 45.628 ± 0.264 | 45.546 ± 0.269 |
| PUFA | 47.655 ± 0.287 | 48.749 ± 0.301 | 48.402 ± 0.311 | 47.783 ± 0.550 | 47.851 ± 0.268 | 47.394 ± 0.408 | 22.020 ± 0.516 | 22.418 ± 0.381 | 22.326 ± 0.387 | 22.688 ± 0.464 | 22.345 ± 0.292 | 21.669 ± 0.360 | 31.183 ± 0.221 | 31.601 ± 0.141 | 31.422 ± 0.223 | 31.541 ± 0.202 | 30.284 ± 0.217 | 30.228 ± 0.327 |
| n-6 PUFA | 44.040 ± 0.318 | 45.588 ± 0.305 | 45.197 ± 0.320 | 44.621 ± 0.572 | 44.583 ± 0.263 | 44.059 ± 0.404 | 15.905 ± 0.353 | 16.446 ± 0.191 | 16.595 ± 0.226 | 16.723 ± 0.231 | 16.278 ± 0.202 | 15.951 ± 0.237 | 10.471 ± 0.105 | 10.481 ± 0.126 | 10.443 ± 0.170 | 10.539 ± 0.127 | 10.458 ± 0.117 | 10.525 ± 0.107 |
| n-3 PUFA | 2.891 ± 0.062 | 2.834 ± 0.047 | 2.900 ± 0.046 | 2.923 ± 0.051 | 2.781 ± 0.060 | 2.788 ± 0.049 | 5.902 ± 0.321 | 5.762 ± 0.323 | 5.531 ± 0.270 | 5.749 ± 0.287 | 5.849 ± 0.336 | 5.502 ± 0.295 | 18.881 ± 0.119 | 19.390 ± 0.062 | 19.298 ± 0.161 | 19.388 ± 0.129 | 18.070 ± 0.139 | 17.955 ± 0.338 |
| n-3/n-6 PUFA | 0.066 ± 0.002 | 0.062 ± 0.001 | 0.064 ± 0.001 | 0.066 ± 0.002 | 0.063 ± 0.001 | 0.063 ± 0.001 | 0.372 ± 0.021 | 0.351 ± 0.020 | 0.333 ± 0.016 | 0.343 ± 0.015 | 0.361 ± 0.024 | 0.346 ± 0.020 | 1.804 ± 0.014 | 1.852 ± 0.025 | 1.852 ± 0.037 | 1.841 ± 0.022 | 1.729 ± 0.021 | 1.707 ± 0.037 |

*i* = iso, *ai* = anteiso, SFA = saturated fatty acid, MUFA = monounsaturated fatty acid, PUFA = polyunsaturated fatty acid, n-3/n-6 PUFA = ratio of n-3 PUFA total to n-6 PUFA total.
